# Probiotic *Streptococcus salivarius* K12 Alleviates Radiation-Induced Oral Mucositis in Mice

**DOI:** 10.3389/fimmu.2021.684824

**Published:** 2021-06-04

**Authors:** Yan Wang, Jiatong Li, Haonan Zhang, Xin Zheng, Jiantao Wang, Xiaoyue Jia, Xian Peng, Qian Xie, Jing Zou, Liwei Zheng, Jiyao Li, Xuedong Zhou, Xin Xu

**Affiliations:** ^1^ State Key Laboratory of Oral Diseases & National Clinical Research Center for Oral Diseases & Department of Pediatric Dentistry, West China Hospital of Stomatology, Sichuan University, Chengdu, China; ^2^ State Key Laboratory of Oral Diseases & National Clinical Research Center for Oral Diseases & Department of Cariology and Endodontics, West China Hospital of Stomatology, Sichuan University, Chengdu, China; ^3^ State Key Laboratory of Biotherapy, Department of Lung Cancer Center and Department of Radiation Oncology, West China Hospital, Sichuan University, Chengdu, China; ^4^ State Key Laboratory of Oral Diseases & National Clinical Research Center for Oral Diseases, West China Hospital of Stomatology, Sichuan University, Chengdu, China; ^5^ Department of Endodontics, College of Dentistry, University of Illinois at Chicago, Chicago, IL, United States; ^6^ Clinical Research Center for Oral Diseases of Sichuan Province, Chengdu, China

**Keywords:** *Streptococcus salivarius* K12, radiotherapy, oral mucositis, dysbiosis, probiotics

## Abstract

**Background:**

Oral mucositis is the most common oral complication of cancer patients receiving radiotherapy and/or chemotherapy, leading to poor quality of life. Limitations of the current interventions on radiation-induced oral mucositis (RIOM) urge the development of novel therapeutics. Here, we evaluated the treatment outcome of probiotic *Streptococcus salivarius* K12 on RIOM mice, and oral microbiota that is associated with the progress of RIOM was further investigated.

**Methods:**

An experimental RIOM mouse model was established, and *S. salivarius* K12 was applied to the mouse oral cavity daily. Histological analyses were performed to evaluate the severity of oral mucositis and the treatment outcome of *S. salivarius* K12. The oral microbiota of mice was further analyzed by 16S rRNA sequencing, microbial culture and qPCR.

**Results:**

Irradiation induced conspicuous mucositis in the oral cavity of mice. *S. salivarius* K12 treatment was beneficial for the healing of RIOM, as reflected by reduced ulcer size, increased basal layer epithelial cellularity and mucosal thickness, and elevated epithelial proliferation and attenuated apoptosis. RIOM mice presented significant oral microbial dysbiosis, with an overgrowth of oral anaerobes. *S. salivarius* K12 treatment reconstituted the oral microbiota and decreased the abundance of oral anaerobes of RIOM mice. In addition, *S. salivarius* K12 treatment inhibited NI1060 in *Pasteurella* genus and downregulated the expression of nitrate reductase.

**Conclusions:**

*S. salivarius* K12 treatment can alleviate RIOM and reconstituted the dysbiotic oral microbiota in mice. *S. salivarius* K12 may represent a promising adjuvant treatment to improve the quality of life of cancer patients receiving radiotherapy.

## Introduction

Oral mucositis, characterized by inflammation and mucosal damage of oral mucosa, is the most common oral complication of cancer patients receiving radiotherapy and/or chemotherapy. The incidence of oral mucositis is almost 100% in head and neck cancer patients receiving radiotherapy (1, 2). 85% of patients receiving intensive chemotherapy for hematopoietic stem cell transplant also develop oral mucositis (3). Oral mucositis induced by radiotherapy/chemotherapy can cause pain, dysphagia and malnutrition, seriously affecting the quality of life of patients and interrupting anti-cancer treatment. Till now, the management of oral mucositis is still challenging. Interventions include growth factors, antibiotics, chlorhexidine, cryotherapy, low-level laser therapy and anti-inflammatory agents, but with limited efficacy (4). At present, only keratinocyte growth factor-1 (palifermin) has been approved by the US Food and Drug Administration to mitigate oral mucositis in a very limited segment of the at-risk population (5). Hence, there is still a need for the development of novel therapeutics for the better management of oral mucositis.

The pathogenesis of mucositis induced by radiotherapy/chemotherapy has been suggested in previous studies (6, 7). Sonis et al. depicted the development of oral mucositis and intestine mucositis as a dynamic process including five stages: initiation, primary damage response, signal amplification, ulceration and healing (6). Recent studies have suggested the role of oral microbiota in the development and progression of oral mucositis (8). Microbial dysbiosis, invasion and colonization of oral mucosa were involved in the pathophysiology of oral mucositis (9, 10). Pathogens contributed to the development of oral mucositis by activating inflammatory responses through pathogen-associated molecular patterns (PAMPs), which bind to pattern recognition receptors (PRRs), subsequently activate NF-ĸB and induce the release of pro-inflammatory cytokines (10).

Probiotics which consist of beneficial viable bacteria and bacterial components, have shown various beneficial effects on human health, particularly *via* modulating the disease-related dysbiotic microbiota (11, 12). *Streptococcus salivarius* is a commensal bacterium in the oral cavity, and the *S. salivarius* K12 strain that was originally isolated from the oral cavity of a healthy child, has been well recognized as an oral probiotic being used for the treatment of multiple oropharyngeal pathogen-related diseases including oral candidiasis, pharyngitis, and halitosis (13–15). *S. salivarius* K12 has a regulatory effect on oral microflora (15, 16), likely due to its potent production of bacteriocin-like inhibitory substances (BLISs) including Lanibiotics salivaricin A and salivaricin B. The production of BLISs contributes to the competitiveness of *S. salivarius* K12 over pathogens and thus benefits the oropharyngeal health (17). The regulatory effects of *S. salivarius* K12 on oral microbiota imply its potential use in the treatment of RIOM. Hence, we hypothesize that radiation can alter oral microbiota and predispose the host to oral mucositis, and *S. salivarius* K12 can alleviate mucositis by modulating the oral microbiota. To validate this hypothesis, we established a RIOM mouse model, and analyzed the ecological impact of radiation on the oral microbiota. In addition, beneficial effects of *S. salivarius* K12 on the healing of oral mucositis were further evaluated.

## Materials and Methods

### Radiation-Induced Oral Mucositis Mouse Model

Seven-week-old male BLAB/c mice were purchased and housed under specific pathogen-free conditions. All animal procedures in this study were approved by Ethics Committee of State Key Laboratory of Oral Diseases, Sichuan University, Chengdu, China. This study conformed to the “Animal Research: Reporting of *In Vivo* Experiments” guidelines for preclinical studies.

After one week of environment acclimation, the mice were randomly divided into three groups (N=11 per group, 5 for macroscopic analyses and 6 for histological analyses and microbial analyses): irradiation-free (control), irradiation+Saline (IR+Saline) and irradiation+ *S. salivarius* K12 (IR+K12). The mice were immobilized for irradiation with chloral hydrate. The technique and set-up for head-only radiation treatment in mice were modified based on previously published studies (18). Custom-made lead shields were used for mice to limit the radiation to the heads. Mice received a high dose, single fractionated 28Gy X-ray radiation directly to their head region at rate of 3.5 Gy/min. Except for the control group, the mice received X-ray radiation. Post-irradiation mice recovered on heated pad before return to vivarium.

### Treatment With Probiotics

Probiotics solution was prepared by dissolving probiotics tablets, which contain 1×10^10^ CFU of viable bacteria per tablet according to the manufacturer’s instruction (NOW Foods, USA), in sterilized saline to a concentration of 1X10^10^ CFU/ml of *S. salivarius* K12. 100 *µ*l probiotic solution (1×10^9^ CFU of *S. salivarius* K12 per day) was applied to the oral cavity of mouse using a micropipette from day -3 to day 8. After administration, food and water were unavailable within the next 30 min to keep the probiotics in mouth as long as possible. The mice in IR+Saline group were treated with saline as placebo. All mice were sacrificed at day 9. The microbial samples were taken before sacrifice.

### Macroscopic and Histological Analyses

Mice were sacrificed and the whole tongue was then removed from oral cavity. Excised tongues were stained with 0.05% toluidine blue to visualize the ulceration (19). Excised tongues of mice were stained with 0.05% toluidine blue for 10 min and rinsed with 10% acetic acid for 1 min to visualize the ulceration (19). Ulcers were visible as a deep blue color after staining. Then, the percentages of stained area to whole area of tongue surfaces were measured by pixels on Image J software to determine the ulcer area.

Paraffin sections were prepared for histological analyses. For staining based on hematoxylin and eosin (H&E), cell proliferation (PCNA), or apoptotic cells (TUNEL), specimens were fixed in 4% paraformaldehyde and embedded in paraffin. After deparaffinization, sections were stained with hematoxylin and eosin to confirm the histologic changes. To measure the epithelial thickness, two sites in five randomly chosen hematoxylin and eosin-stained sections (six specimens/group) were measured. The basal layer cellularity was measured by counting the absolute number of cells at basal layer at the area of interest in five randomly chosen sections (six specimens/group). To confirm the tissue-regenerative activity (cell proliferation) of damaged tissues, sections were stained with rat monoclonal anti-mouse PCNA (1:100; Abcam, Cambridge, UK). For the apoptosis assay, TUNEL (terminal deoxynucleotidyl transferase dUTP nick and labeling) staining was performed with a TUNEL kit (Beyotime, China) according to manufacturer’s protocol to detect apoptotic cells. Slides were mounted with coverslips using DAPI Fluoromount-G (Southern Biotech, China). To quantify of cell proliferation (PCNA) and apoptotic activities, the absolute number of positive cells at the area of interest in five randomly chosen sections (six specimens/group) were counted.

### 16S rRNA Sequencing

Bacterial DNA was extracted from oral samples from the mice. DNA library was prepared with uniquely barcoded primer targeting the V3/4 region of the 16S rRNA as described previously (20). The library construction and sequencing data analyses were performed as previously described (21). The oral swabs from the mice were sequenced at Majorbio Co. (Shanghai, China). 338F (5’- ACTCCTACGGGAGGCAGCAG-3’) and 806R (5’- GGACTACHVGGGTWTCTAAT-3’) primers were used to amplify the V3/V4 region of 16S rDNA. Barcoded 16S rDNA amplicon sequencing was performed through Illumina MiSeq platform. Sequences were trimmed using Trimmomatic (22) based on quality scores of 20, and pair-end reads were merged into longer reads by FLASH (23). Unqualified sequences, too short or contained ambiguous residues, were removed. Operational taxonomic units (OTUs) were clustered using Usearch version 7.0 (http://drive5.com/uparse/) at the 97% similarity level, and final OTUs were generated based on the clustering results. All sequencing data were uploaded to NCBI SRA database with an accession number SRP276563.

The pre-processed sequencing data was further analyzed with the following statistical methods. (1) Alpha diversity analysis was based on Shannon index. (2) PCoA (principal coordinates analysis) was used to compare the beta diversity within groups. Two non-parametric analyses for multivariate data, multivariate analysis of variance (Adonis) and analysis of similarities (ANOSIM) using distance matrices, were used to examine the community difference within groups. (3) Taxonomic annotations were assigned to each OTU’s representative sequence by blasting with the oral “CORE” reference database. The relative abundances of bacterial taxa at genus levels were analyzed and compared. All analyses were performed with I-Sanger online tools (http://www.i-sanger.com/).

### Quantitative Real-Time PCR

The qPCR amplification was performed on a StepOnePlus™ Real-Time PCR System (Applied Biosystems). For quantification of total bacterial load, the reaction mixture (25 μl) contained SYBR^®^ Premix Ex Tag II (Takara Bio), microbial genomic DNA (2 μl), and forward and reverse primers (10 μM each). Threshold cycle (CT) values were determined, and relative ratio of 16S and 18S was calculated based on the 2^–ΔΔCT^ method (24). NI1060 was quantified with the protocol described previously (25). For quantification of the expression level of *napA*, microbial RNA was extracted with TriZol Reagent (Invitrogen) according to the manufacturer’s instructions. Reverse transcription of RNA into cDNA was performed with the PrimeScript RT Reagent Kit with gDNA Eraser (Takara Bio). The expression levels of *napA* were measured with that of the control group as control. The sequence of the primers (16S, 18S and *napA*) used referred to the previous study (24).

### Anaerobic Bacteria Cultivation

Oral mucosa of mice was swabbed for 30 s and the swabs were then inserted into Eppendorf tubes containing 100 *µ*L of Wilkins-Chalgren medium (Oxoid). The samples were serially diluted and plated on blood agar for two days under anaerobic conditions at 37°C. Colonies were enumerated to determine the colony-forming units (CFUs) of total cultivatable oral anaerobic bacteria (24).

### Statistical Analysis

For 16S rRNA sequencing data, statistical analyses were performed with I-Sangers online tools (http://www.i-sanger.com/) (25, 26). The differences in beta diversity (revealed by PCoA) within groups were compared; the alpha diversity data and genus-level microbial composition data were analyzed by Wilcoxon rank-sum test for two group comparison and Kruska-Wallis H test with the Dunn’s test for three group comparisons. All other data were statistically analyzed by GraphPad Prism 6. Differences between groups were analyzed by one-way analysis of variance test followed by Tukey’s test. A two-tailed P<0.05 was considered significant.

## Results

### *S. salivarius* K12 Ameliorates Radiation-Induced Oral Mucositis in Mice

**Figure 1:**
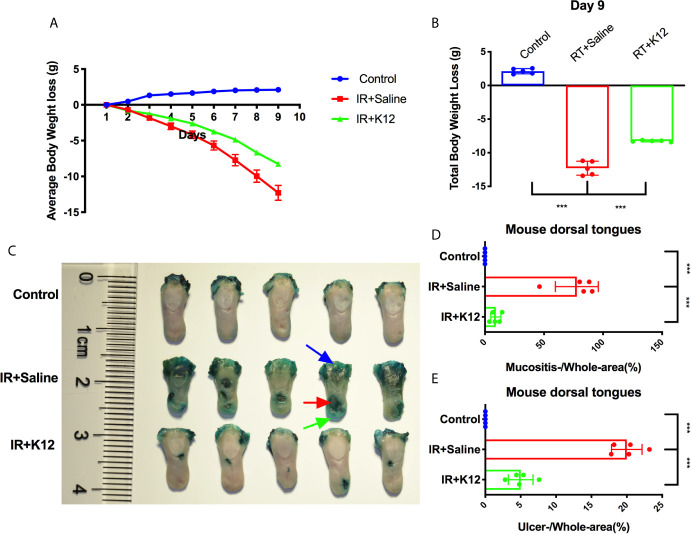
*Streptococcus salivarius* K12 alleviates body weight loss and reduces tongue ulcer area in RIOM mice. **(A)** Average body weight loss. **(B)** Total body weight loss. **(C)** Toluidine blue staining of harvested tongues. The area of mucositis with (red arrow) and without ulcer (green arrow) was stained blue. Blue arrow: staining at the site of incision to remove tongue. **(D)** Quantitative analyses of mucositis area (mucositis+ulcer/whole surface area). **(E)** Quantitative analyses of ulcer area (ulcer/whole surface area). Data are presented as mean ± SD. ***P < 0.001. N = 5 per group.

**Figure 2:**
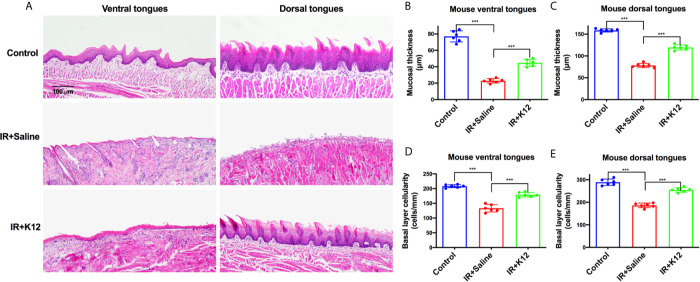
*Streptococcus salivarius* K12 promotes RIOM healing in mice. **(A)** Representative images of H&E staining indicating the integrity of lingual mucosa (Scale bar, 100 µm). **(B, C)** Quantitative analysis of mucosal thickness of ventral tongues and dorsal tongues, respectively. **(D, E)** Basal layer epithelial cellularity of ventral tongues and dorsal tongues, respectively. Data are presented as mean ± SD. N=6 per group. One-way ANOVA test followed by Tukey’s test. ***P<0.001.

The body weights of mice that received irradiation decreased sharply, while *S. salivarius* K12 treatment alleviated the body weight loss (**Figure 1A**). The total body weight loss of *S. salivarius* K12 treatment group (-8.33g) was significantly less than that of irradiated mice (-12.05g) on the 9^th^ day after irradiation (**Figure 1B**). In the oral cavity of irradiated mice, conspicuous mucositis, particularly on the lingual mucosa was observed as reflected by toluidine blue staining (**Figure 1C**). Topical application of *S. salivarius* K12 significantly reduced the severity of oral mucositis in irradiated mice (**Figure 1C–E**). Specifically, the relative area of mucositis including ulcers was significant reduced in the IR+K12 group (9.03%) as compared to that in the IR+Saline group (77.42%) (**Figure 1D**). The relative ulcer area in tongues of the *S. salivarius* K12-treated mice (5.02%) was also significantly lower than the IR+Saline group (20.21%) (**Figure 1E**). H&E staining showed conspicuous mucosal hypoplasia and ulceration in the tongue of irradiated mice, while *S. salivarius* K12 treatment partially restored the integrity of the lingual mucosa (**Figure 2A**). Topical use of *S. salivarius* K12 also significantly increased mucosal thickness (**Figures 2B, C**) and basal layer epithelial cellularity in both ventral and dorsal tongues (**Figures 2D, E**).

**Figure 3:**
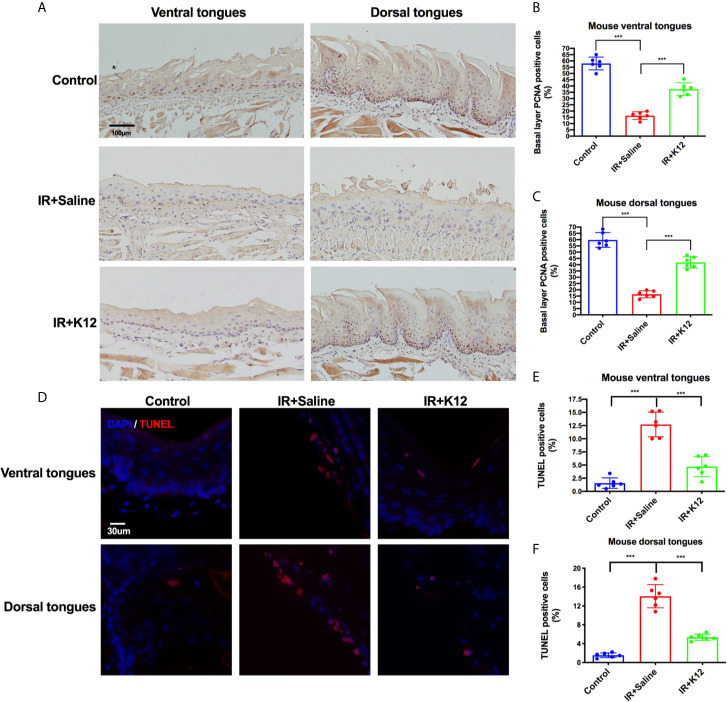
*Streptococcus salivarius* K12 promoted the proliferation of mouse tongue basal layer cells and reduced apoptosis of mouse tongue mucosal cells. **(A)** Representative microscopic images of mouse tongues PCNA staining (Scale bar, 100 µm). **(B)** Percentage of basal layer PCNA positive cells in mouse ventral tongues. **(C)** Percentage of basal layer PCNA positive cells in mouse dorsal tongues. **(D)** Representative microscopic images of TUNEL staining on mouse tongues (Scale bar, 30 µm). **(E)** Percentage of TUNEL-positive cells in mouse ventral tongues. **(F)** Percentage of TUNEL-positive cells in mouse dorsal tongues. Data are presented as mean ± SD. N=6 per group. One-way ANOVA test followed by Tukey’s test. ***P < 0.001.

Further immunohistology showed that irradiation attenuated the proliferation of basal layer epithelial cells and induced significant apoptosis in both ventral and dorsal tongue of RIOM mice as compared to the irradiation-free controls (**Figure 3**). *S. salivarius K12* treatment significantly rescued this pathology, as increased number of PCNA^+^ proliferative cells in the baser layer epithelium (**Figures 3A–C**) and decreased number of TUNEL^+^ apoptotic cells (**Figures 3D–F**) were observed in both dorsal and ventral tongues of the IR+K12 group compared to the IR+Saline group.

### *S. salivarius* K12 Modulates Oral Microbiota in RIOM Mice

**Figure 4:**
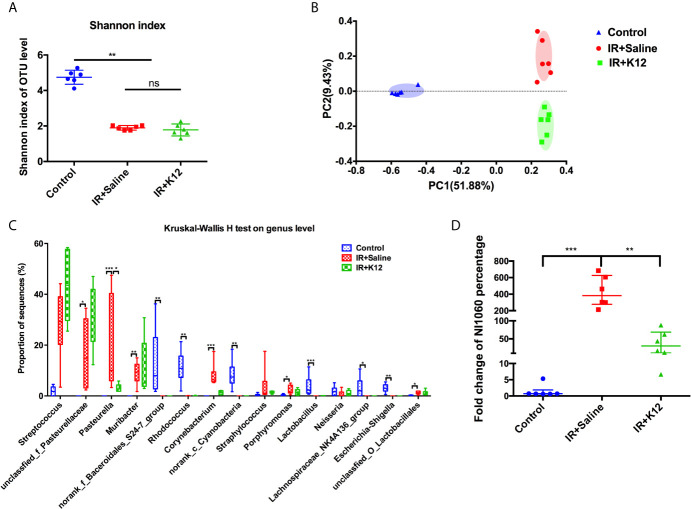
*Streptococcus salivarius* K12 modulates oral microbiota in RIOM mice. **(A)** The alpha diversity of oral microbiota. **(B)** Principal coordinate analysis (PCoA) of oral microbiota based on Bray-Curtis distance. **(C)** Prevalent genus with significant difference in abundance. Values are presented as median, interquartile range, minimum, and maximum. Kruska-Wallis H test with *post hoc* tests applying the Dunn’s test for multiple comparisons. **(D)** qPCR quantification of NI1060 (mean ± SD). One-way ANOVA test followed by Tukey’s test. N=6 per group. ns, not significant. *P < 0.05, **P < 0.01, ***P < 0.001.

16S rRNA sequencing data showed that the oral cavity of RIOM mice harbored a microbiota with lower alpha diversity relative to the irradiation-free controls (**Figure 4A**). Principal coordinates analysis (PCoA) based on Bray-Curtis distance showed that the oral microbiota of RIOM was distinct from that of irradiation-free controls (**Figure 4B**), indicating an altered microbial structure. Further analysis of the top 15 abundant bacterial taxa revealed genus-level differences between RIOM mice and irradiation-free controls (**Figure 4C**). The oral microbiota of RIOM mice had significantly increased abundance of *unclassified_f_Pasteurellaceae*, *Pasteurella*, *Muribacter*, *Corynebacterium* and *unclassified_O_Lactobacillales* and lower levels of *norank_f_Bacteroidales_S24-7_group*, *Rhodococcus*, *norank_c_Cyanobacteria*, *Lactobacillus*, Lachnospiraceae_NK4A136_group and *Escherichia-Shigella* as compared to the irradiation-free controls.

The oral microbiota of RIOM mice with/without *S. salivarius* K12 treatment showed no difference in alpha diversity (**Figure 4A**). PCoA based on Bray-Curtis distance revealed differential clustering of the oral microbiota among the IR+Saline, IR+K12, and irradiation-free controls, suggesting partial reconstitution of microbial structure after *S. salivarius K12* treatment (**Figure 4B**). More importantly, *S. salivarius K12* treatment altered the microbial composition of RIOM mice. An enrichment of *Pasteurella* was observed in IR+Saline group as compared to the irradiation-free controls, and *S. salivarius K12* treatment reduced the amount of *Pasteurella* in the RIOM mice (**Figure 4C**). Further species-specific qPCR analysis showed that a periodontitis-associated pathogen NI1060 in the *Pasteurella* genus was significantly enriched in IR+Saline group, and *S. salivarius* K12 treatment significantly reduced its abundance in the oral cavity of RIOM mice (**Figure 4D**).

### *S. salivarius* K12 Suppresses the Overgrowth of Oral Anaerobes in RIOM Mice

**Figure 5:**
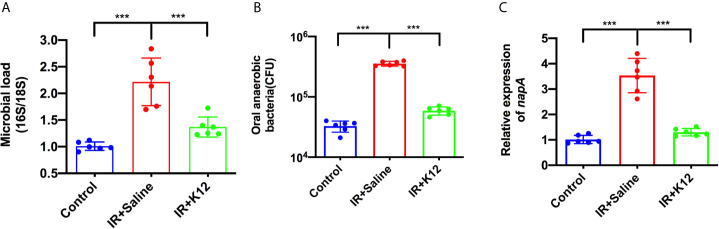
*Streptococcus salivarius* K12 suppresses the overgrowth of oral anaerobes in RIOM mice. **(A)** Total bacteria in oral swabs were quantified by qPCR normalized as 16S/18S rRNA. **(B)** Total cultivable oral anaerobes in oral swabs. **(C)** Relative expression levels of nitrate reductase *napA* gene of oral microbiota. Data are presented as mean ± SD. N=6 per group. One-way ANOVA test followed by Tukey’s test. ***P < 0.001.

Microbial samples from the oral swabs were further analyzed to investigate the effect of *S. salivarius* K12 treatment on the oral microbiota of RIOM mice. The oral cavity of IR+Saline mice was colonized with twice the number of bacteria as compared to the irradiation-free controls; while *S. salivarius K12* treatment significantly reduced the overall microbial load (**Figure 5A**). More importantly, the IR+Saline group presented an increased amount of cultivated anaerobic bacteria as compared to the irradiation-free controls, and *S. salivarius K12* treatment significantly reduced the amount of cultivated anaerobic bacteria in RIOM mice (**Figure 5B**). Consistently, an elevated expression of gene encoding nitrate reductase (*napA*) was observed in the oral microbiota of RIOM mice as compared to that of irradiation-free controls, and *S. salivarius* K12 treatment significantly downregulated *napA* expression (**Figure 5C**).

## Discussion

Radiotherapy is one of the most commonly used treatment modalities for head and neck cancers, but with frequent complications as oral mucositis. Limited evidence has suggested the involvement of oral microbes in the development of oral mucositis in this context (8, 27, 28). In the current study, we demonstrated that radiation caused oral microbial dysbiosis of RIOM mice. More importantly, topical use of probiotic *S. salivarius K12* ameliorated oral mucositis in RIOM mice by modulating the oral microbiota, representing a promising approach to the management of RIOM.

The pathogenesis of radiation-induced oral mucositis remains undefined, and the complex interaction between microbiota and host has been suggested (9, 10, 29). Recently, increasing evidence suggests that microbiota has played an important role in the pathogenesis of mucositis (29, 30). Hu et al. analyzed the supragingival plaque of eight nasopharyngeal carcinoma (NPC) patients receiving head and neck radiotherapy and found that the relative abundance of core microorganisms changed dynamically during radiotherapy (27, 28). Zhu et al. revealed that bacterial community structure altered progressively in NPC patients during radiotherapy, accompanied with a marked increase of certain Gram-negative bacteria. Patients who eventually developed severe oral mucositis harbored a higher abundance of *Actinobacillus* during the phase of erythema-patchy mucositis (8). Reyes-Gibby et al. found that changes in the abundance of genera, including 1) *Cardiobacterium* and *Granulicatella* at the baseline; 2) *Prevotella*, *Fusobacterium* and *Streptococcus* immediately before the development of oral mucositis; and 3) *Megasphaera* and *Cardiobacterium* immediately before the development of severe oral mucositis, over the course of treatment in the patients with squamous cell carcinoma of the head and neck were associated with the onset of severe oral mucositis (31). These data suggest the involvement oral microbial dysbiosis in the development of RIOM.

*S. salivarius* K12 was first isolated from the throat of a healthy child, and is currently used as a probiotic for the treatment oral malodor, oral candidiasis, secretory otitis media and pharyngotonsillitis (14, 15, 32–35). Given the presence of oral microbial dysbiosis after irradiation, we used *S. salivarius* K12 to treat the RIOM mice. Our results showed that *S. salivarius* K12 could modulate oral microbiota, and effectively alleviated RIOM, as reflected by a significant reduction of ulceration, increased thickness of tongue mucosa and the density of basal cells, enhanced basal cell proliferation, and attenuated apoptosis. Although few studies have demonstrated beneficial effects of probiotics in the treatment/prevention of oral mucositis, satisfactory outcomes have also been reported by others using probiotics to treat intestinal mucositis induced by radiotherapy/chemotherapy. Kato et al. found that *Bifidobacterium bifidum* G9-1 could improve the microbial dysbiosis and thus ameliorated 5-Fluorouracil-induced intestinal mucositis in mice (11). Consistently, another study found that administration of a probiotic mixture DM#1, which includes four probiotic strains as *Bifidobacterium breve* DM8310, *Lactobacillus acidophilus* DM8302, *Lactobacillus casei* DM8121 and *S. thermophiles* DM8309, ameliorated 5-Fluorouracil-induced intestinal mucositis *via* the reestablishment of microbial homeostasis and regulation of the TLR2/TLR4 signaling pathway (36). *Lactobacillus rhamnosus* GG also shows a protective effect on the intestinal epithelium from radiation injury, possibly through the release of lipoteichoic acid that activates the radioprotective TLR2 (37).

In addition to the shift of oral microbiota after irradiation, we also observed an increased microbial load and overgrowth of anaerobic bacteria in the oral cavity of RIOM mice, along with an elevated expression of nitrate reductase gene (*napA*). Consistently, Kato et al. reported an elevated amount of anaerobic bacteria in mice with 5-fluorouracil-induced intestinal mucositis (11). Nassar et al. also reported a higher expression levels of *napA* in Gas6−/− mice, which harbored dysbiotic microbiota with expansion of anaerobic bacteria (24). NapA, as an important nitrate reductase, plays an important role in the growth of anaerobic pathogenic bacteria. By expressing nitrate reductase, the anaerobic bacteria can use electron acceptors generated as a byproduct of inflammation to support their growth by anaerobic respiration (24, 38). Lipopolysaccharides (LPS) produced by Gram-negative anaerobes could induce inflammatory responses by activating toll-like receptor 4 signaling pathway (39), which may contribute to the development of oral mucositis. In the current study, *S. salivarius* K12 treatment reduced the number of anaerobic bacteria and downregulated the expression of *napA* in RIOM mice. In addition, *S. salivarius* K12 treatment reduced the abundance of bacterial genera including *Pasteurella, Corynebacterium*, *Porphyromonas*, and *Staphylococcus*. Intriguingly, an increase of NI1060 in the *Pasteurella* genus was observed in RIOM mice. NI1060 is an inflammation-related bacterium identified in murine ligature-induce periodontitis (40). The genomic sequencing of NI1060 revealed its possible virulence genes involved in lipooligosaccharide synthesis, adhesins and bacteriotoxic proteins, which were potentially important for host adaption and induction of dysbiosis through bacterial competition and pathogenicity (41). Our recent study on periodontitis also suggests the involvement of this bacteria in periodontitis (25). The enrichment of NI1060 in the oral cavity of mice receiving radiation suggests a possible involvement of this pathogenic bacteria in the development of RIOM. Although more studies are still needed, this bacterium may provide a potential target for the treatment/prevention of RIOM.

Some cautions should be taken when interpreting data from the current study. Firstly, the RIOM mouse model was established using a single high-dose irradiation referring to a previous study (18). Nevertheless, conventional radiotherapy for cancer patients consists of low fractionated X-ray doses lasting for weeks in most cases (42). Since single high-dose irradiation and low-dose fractionated irradiation could exert different effects on oral mucosa and oral microbiota, future studies using animal models closer to the clinical radiation regimens are warranted. Secondly, we only sampled oral microbial samples on day 9 post irradiation because overt ulceration in mouse tongue mucosa peaked 9 days after irradiation. Whether *S. salivarius* K12 can accelerate the restoration of microbial dysbiosis still needs dynamic sampling after irradiation. Thirdly, although *S. salivarius* K12 showed effectiveness in the treatment of RIOM and the inhibition of oral anaerobes in the current study, whether this probiotic strain can directly act on tissue inflammation other than competing oral anaerobes to promote the healing of RIOM still needs further investigations. It should be noted that changes in commensal bacteria took place at the early stage of radiotherapy (27, 28), suggesting that microbial alteration could possibly be an initiating factor rather than a consequence of oral mucositis. A recent systematical review including five clinical studies of 435 patients has indicated that probiotics may help reduce the incidence and mitigate the severity of cancer therapy-induced oral mucositis (43). However, as the selection and combination of probiotics, application method and target population vary among these studies, more evidence is still needed to justify the clinical application of probiotic in this scenario. In addition, the differed host responses of probiotics warrant customized probiotic interventions on patients receiving varying anti-cancer treatment modalities (44). It is also noteworthy that the use of probiotics may cause invasive infection in patients with compromised immunity (45). More work is still needed to translate the application of probiotics to the management of RIOM in the future.

In summary, our data show that probiotic *S. salivarius* K12 can modulate oral microbiota and ameliorate radiation-induced oral mucositis in a RIOM mice model. *S. salivarius* K12 as a probiotic represents a promising therapeutic against RIOM. Since probiotics have been proposed as a potential approach to the management of radiotherapy/chemotherapy-induced mucositis (46), *S. salivarius* K12 as an oral probiotic represents a promising adjuvant treatment to improve the quality of life of cancer-patients receiving radiotherapy.

## Data Availability Statement 

The datasets presented in this study can be found in online repositories. The names of the repository/repositories and accession number(s) can be found below: https://www.ncbi.nlm.nih.gov/, SRP276563.

## Ethics Statement

The animal study was reviewed and approved by Ethics Committee of State Key Laboratory of Oral Diseases, Sichuan University, Chengdu, China.

## Author Contributions

YW, JTL, XiZ and XX contributed to conception, design, data acquisition, analysis, and interpretation, drafted and critically revised the manuscript. HZ, XuZ and JW contributed to data acquisition and interpretation, critically revised the manuscript. XJ, XP, JZ, LZ and JYL contributed to data interpretation, critically revised the manuscript. All authors contributed to the article and approved the submitted version.

## Funding

This study was supported by the National Natural Science Foundation of China (81600864, 81771099), and a research grant from the Science and Technology Department of Sichuan Province (2018SZ0121).

## Conflict of Interest

The authors declare that the research was conducted in the absence of any commercial or financial relationships that could be construed as a potential conflict of interest.

## References

[1] Maria OM Eliopoulos N and Muanza T. Radiation-Induced Oral Mucositis. Front Oncol (2017) 7:89. 10.3389/fonc.2017.00089 28589080 PMC5439125

[2] Vera-Llonch M Oster G Hagiwara M and Sonis S. Oral Mucositis in Patients Undergoing Radiation Treatment for Head and Neck Carcinoma. Cancer (2006) 106(2):329–36. 10.1002/cncr.21622 16342066

[3] Chaudhry HM Bruce AJ Wolf RC Litzow MR Hogan WJ Patnaik MS. The Incidence and Severity of Oral Mucositis Among Allogeneic Hematopoietic Stem Cell Transplantation Patients: A Systematic Review. Biol Blood Marrow Transplant (2016) 22(4):605–16. 10.1016/j.bbmt.2015.09.014 26409924

[4] Hong CHL Gueiros LA Fulton JS Cheng KKF Kandwal A Galiti D. Systematic Review of Basic Oral Care for the Management of Oral Mucositis in Cancer Patients and Clinical Practice Guidelines. Support Care Cancer (2019) 27(10):3949–67. 10.1007/s00520-019-04848-4 31286232

[5] Cinausero M Aprile G Ermacora P Basile D Vitale MG Fanotto V. New Frontiers in the Pathobiology and Treatment of Cancer Regimen-Related Mucosal Injury. Front Pharmacol (2017) 8:354. 10.3389/fphar.2017.00354 28642709 PMC5462992

[6] Sonis ST. The Pathobiology of Mucositis. Nat Rev Cancer (2004) 4(4):277–84. 10.1038/nrc1318 15057287

[7] Vanhoecke B De Ryck T Stringer A Van de Wiele T and Keefe D. Microbiota and Their Role in the Pathogenesis of Oral Mucositis. Oral Dis (2014) 21(1):17–30. 10.1111/odi.12224 24456144

[8] Zhu XX Yang XJ Chao YL Zheng HM Sheng HF Liu HY. The Potential Effect of Oral Microbiota in the Prediction of Mucositis During Radiotherapy for Nasopharyngeal Carcinoma. EBioMedicine (2017) 18:23–31. 10.1016/j.ebiom.2017.02.002 28216066 PMC5405060

[9] Sonis ST. The Chicken or the Egg? Changes in Oral Microbiota as Cause or Consequence of Mucositis During Radiation Therapy. EBioMedicine (2017) 18:7–8. 10.1016/j.ebiom.2017.03.017 28330600 PMC5405157

[10] Vanhoecke BW De Ryck TR De boel K Wiles S Boterberg T Van de Wiele T. Low-Dose Irradiation Affects the Functional Behavior of Oral Microbiota in the Context of Mucositis. Exp Biol Med (Maywood) (2016) 241(1):60–70. 10.1177/1535370215595467 26202372 PMC4935431

[11] Kato S Hamouda N Kano Y Oikawa Y Tanaka Y Matsumoto K. Probiotic Bifidobacterium Bifidum G9-1 Attenuates 5-Fluorouracil-Induced Intestinal Mucositis in Mice Via Suppression of Dysbiosis-Related Secondary Inflammatory Responses. Clin Exp Pharmacol Physiol (2017) 44(10):1017–25. 10.1111/1440-1681.12792 28590519

[12] Panebianco C Latiano T and Pazienza V. Microbiota Manipulation by Probiotics Administration as Emerging Tool in Cancer Prevention and Therapy. Front Oncol (2020) 10:679. 10.3389/fonc.2020.00679 32523887 PMC7261958

[13] Burton JP Chilcott CN Moore CJ Speiser G Tagg JR. A Preliminary Study of the Effect of Probiotic Streptococcus Salivarius K12 on Oral Malodour Parameters. J Appl Microbiol (2006) 100(4):754–64. 10.1111/j.1365-2672.2006.02837.x 16553730

[14] Di Pierro F Adami T Rapacioli G Giardini N Streitberger C. Clinical Evaluation of the Oral Probiotic Streptococcus Salivarius K12 in the Prevention of Recurrent Pharyngitis and/or Tonsillitis Caused by Streptococcus Pyogenes in Adults. Expert Opin Biol Ther (2013) 13(3):339–43. 10.1517/14712598.2013.758711 23286823

[15] Masdea L Kulik EM Hauser-Gerspach I Ramseier AM Filippi A and Waltimo T. Antimicrobial Activity of Streptococcus Salivarius K12 on Bacteria Involved in Oral Malodour. Arch Oral Biol (2012) 57(8):1041–7. 10.1016/j.archoralbio.2012.02.011 22405584

[16] Cosseau C Devine DA Dullaghan E Gardy JL Chikatamarla A Gellatly S. The Commensal Streptococcus Salivarius K12 Downregulates the Innate Immune Responses of Human Epithelial Cells and Promotes Host-Microbe Homeostasis. Infect Immun (2008) 76(9):4163–75. 10.1128/iai.00188-08 PMC2519405 18625732

[17] Wescombe PA Hale JD Heng NC and Tagg JR. Developing Oral Probiotics From Streptococcus Salivarius. Future Microbiol (2012) 7(12):1355–71. 10.2217/fmb.12.113 23231486

[18] Zhao J Kim K-A De Vera J Palencia S Wagle M Abo A. R-Spondin1 Protects Mice From Chemotherapy or Radiation-Induced Oral Mucositis Through the Canonical Wnt/β-Catenin Pathway. Proc Natl Acad Sci USA (2009) 106(7):2331–36. 10.1073/pnas.0805159106 PMC2650156 19179402

[19] T I Sumita Y Minamizato T Umebayashi M Liu Y Tran SD. Bone Marrow-derived Cell Therapy for Oral Mucosal Repair After Irradiation. J Dent Res (2014) 93(8):813–20. 10.1177/0022034514541124 PMC4293762 24980658

[20] Tong M Jacobs JP McHardy IH and Braun J. Sampling of Intestinal Microbiota and Targeted Amplification of Bacterial 16S rRNA Genes for Microbial Ecologic Analysis. Curr Protoc Immunol (2014) 107:7.41.1–7.41.11. 10.1002/0471142735.im0741s107 25367129 PMC4457454

[21] Zheng X He J Wang L Zhou S Peng X Huang S. Ecological Effect of Arginine on Oral Microbiota. Sci Rep (2017) 7(1):7206. 10.1038/s41598-017-07042-w 28775282 PMC5543048

[22] Bolger AM Lohse M and Usadel B. Trimmomatic: A Flexible Trimmer for Illumina Sequence Data. Bioinformatics (2014) 30(15):2114–20. 10.1093/bioinformatics/btu170 PMC4103590 24695404

[23] Magoč T and Salzberg SL. Flash: Fast Length Adjustment of Short Reads to Improve Genome Assemblies. Bioinformatics (2011) 27(21):2957–63. 10.1093/bioinformatics/btr507 PMC3198573 21903629

[24] Nassar M Tabib Y Capucha T Mizraji G Nir T Pevsner-Fischer M. GAS6 Is a Key Homeostatic Immunological Regulator of Host-Commensal Interactions in the Oral Mucosa. Proc Natl Acad Sci USA (2017) 114(3):E337–46. 10.1073/pnas.1614926114 PMC5255577 28049839

[25] Zheng X Tizzano M Redding K He J Peng X Jiang P. Gingival Solitary Chemosensory Cells Are Immune Sentinels for Periodontitis. Nat Commun (2019) 10(1):4496. 10.1038/s41467-019-12505-x 31582750 PMC6776549

[26] Jia X Jia L Mo L Yuan S Zheng X He J. Berberine Ameliorates Periodontal Bone Loss by Regulating Gut Microbiota. J Dent Res (2019) 98(1):107–16. 10.1177/0022034518797275 30199654

[27] Hu YJ Shao ZY Wang Q Jiang YT Ma R Tang ZS. Exploring the Dynamic Core Microbiome of Plaque Microbiota During Head-and-Heck Radiotherapy Using Pyrosequencing. PloS One (2013) 8(2):e56343. 10.1371/journal.pone.0056343 23437114 PMC3578878

[28] Hu YJ Wang Q Jiang YT Ma R Xia WW Tang ZS. Characterization of Oral Bacterial Diversity of Irradiated Patients by High-throughput Sequencing. Int J Oral Sci (2013) 5(1):21–5. 10.1038/ijos.2013.15 PMC3632764 23538641

[29] Vasconcelos RM Sanfilippo N Paster BJ Kerr AR Li Y Ramalho L. Host-Microbiome Cross-talk in Oral Mucositis. J Dent Res (2016) 95(7):725–33. 10.1177/0022034516641890 PMC4914867 27053118

[30] Wang Y Zhou X Xu X. Oral Microbiota: An Overlooked Etiology for Chemotherapy-Induced Oral Mucositis? J Formos Med Assoc (2015) 114:297–9. 10.1016/j.jfma.2013.10.014 24268725

[31] Reyes-Gibby CC Wang J Zhang L Peterson CB Do KA Jenq RR. Oral Microbiome and Onset of Oral Mucositis in Patients With Squamous Cell Carcinoma of the Head and Neck. Cancer (2020) 126(23):5124–36. 10.1002/cncr.33161 PMC8191575 32888342

[32] Di Pierro F Colombo M Zanvit A Rottoli AS. Positive Clinical Outcomes Derived From Using Streptococcus Salivarius K12 to Prevent Streptococcal Pharyngotonsillitis in Children: A Pilot Investigation. Drug Healthc Patient Saf (2016) 8:77–81. 10.2147/dhps.s117214 27920580 PMC5123729

[33] Gregori G Righi O Risso P Boiardi G Demuru G Ferzetti A. Reduction of Group A Beta-Hemolytic Streptococcus Pharyngo-Tonsillar Infections Associated With Use of the Oral Probiotic Streptococcus Salivarius K12: A Retrospective Observational Study. Ther Clin Risk Manag (2016) 12:87–92. 10.2147/tcrm.s96134 26855579 PMC4725641

[34] Ishijima SA Hayama K Burton JP Reid G Okada M Matsushita Y. Effect of Streptococcus Salivarius K12 on the In Vitro Growth of Candida Albicans and its Protective Effect in an Oral Candidiasis Model. Appl Environ Microbiol (2012) 78(7):2190–9. 10.1128/aem.07055-11 PMC3302625 22267663

[35] Jamali Z Aminabadi NA Samiei M Sighari Deljavan A Shokravi M and Shirazi S. Impact of Chlorhexidine Pretreatment Followed by Probiotic Streptococcus Salivarius Strain K12 on Halitosis in Children: A Randomised Controlled Clinical Trial. Oral Health Prev Dent (2016) 14(4):305–13. 10.3290/j.ohpd.a36521 27508274

[36] Tang Y Wu Y Huang Z Dong W Deng Y Wang F. Administration of Probiotic Mixture DM1 Ameliorated 5-Fluorouracil-Induced Intestinal Mucositis and Dysbiosis in Rats. Nutrition (2017) 33:96–104. 10.1016/j.nut.2016.05.003 27427511

[37] Riehl TE Alvarado D Ee X Zuckerman A Foster L Kapoor V. Lactobacillus Rhamnosus GG Protects the Intestinal Epithelium From Radiation Injury Through Release of Lipoteichoic Acid, Macrophage Activation and the Migration of Mesenchymal Stem Cells. Gut (2019) 68(6):1003–13. 10.1136/gutjnl-2018-316226 PMC7202371 29934438

[38] Winter SE and Bäumler AJ. Dysbiosis in the Inflamed Intestine: Chance Favors the Prepared Microbe. Gut Microbes (2014) 5(1):71–3. 10.4161/gmic.27129 PMC4049941 24637596

[39] Doyle SL and O’Neill LA. Toll-Like Receptors: From the Discovery of NFkappaB to New Insights Into Transcriptional Regulations in Innate Immunity. Biochem Pharmacol (2006) 72(9):1102–13. 10.1016/j.bcp.2006.07.010 16930560

[40] Jiao Y Darzi Y Tawaratsumida K Marchesan JT Hasegawa M Moon H. Induction of Bone Loss by Pathobiont-mediated Nod1 Signaling in the Oral Cavity. Cell Host Microbe (2013) 13(5):595–601. 10.1016/j.chom.2013.04.005 23684310 PMC3721316

[41] Darzi Y Jiao Y Hasegawa M Moon H Núñez G Inohara N. The Genomic Sequence of the Oral Pathobiont Strain Ni1060 Reveals Unique Strategies for Bacterial Competition and Pathogenicity. PloS One (2016) 11(7):e0158866. 10.1371/journal.pone.0158866 27409077 PMC4943601

[42] Deloch L Derer A Hartmann J Frey B Fietkau R and Gaipl US. Modern Radiotherapy Concepts and the Impact of Radiation on Immune Activation. Front Oncol (2016) 6:141. 10.3389/fonc.2016.00141 27379203 PMC4913083

[43] Shu Z Li P Yu B Huang S and Chen Y. The Effectiveness of Probiotics in Prevention and Treatment of Cancer Therapy-Induced Oral Mucositis: A Systematic Review and Meta-Analysis. Oral Oncol (2020) 102:104559. 10.1016/j.oraloncology.2019.104559 31923856

[44] Zmora N Zilberman-Schapira G Suez J Mor U Dori-Bachash M Bashiardes S. Personalized Gut Mucosal Colonization Resistance to Empiric Probiotics is Associated With Unique Host and Microbiome Features. Cell (2018) 174(6):1388–405. 10.1016/j.cell.2018.08.041 30193112

[45] Lalla RV Bowen J Barasch A Elting L Epstein J Keefe DM. Mascc/Isoo Clinical Practice Guidelines for the Management of Mucositis Secondary to Cancer Therapy. Cancer (2014) 120(10):1453–61. 10.1002/cncr.28592 PMC4164022 24615748

[46] Cereda E Caraccia M Caccialanza R. Probiotics and Mucositis. Curr Opin Clin Nutr Metab Care (2018) 21(5):399–404. 10.1097/mco.0000000000000487 29916923

